# Ferroptosis as a Potential Therapeutic Target of Traditional Chinese Medicine for Mycotoxicosis: A Review

**DOI:** 10.3390/toxics11040395

**Published:** 2023-04-21

**Authors:** Wenli Ding, Luxi Lin, Ke Yue, Yanfeng He, Bowen Xu, Aftab Shaukat, Shucheng Huang

**Affiliations:** 1 College of Veterinary Medicine, Henan Agricultural University, Zhengzhou 450046, China; dingwenli3011@163.com (W.D.);; 2 National Center for International Research on Animal Genetics, Breeding and Reproduction (NCIRAGBR), Huazhong Agricultural University, Wuhan 430070, China

**Keywords:** aflatoxin B1, Chinese herbs, ferroptosis, lipid peroxidation, mycotoxin, iron metabolism

## Abstract

Mycotoxin contamination has become one of the biggest hidden dangers of food safety, which seriously threatens human health. Understanding the mechanisms by which mycotoxins exert toxicity is key to detoxification. Ferroptosis is an adjustable cell death characterized by iron overload and lipid reactive oxygen species (ROS) accumulation and glutathione (GSH) depletion. More and more studies have shown that ferroptosis is involved in organ damage from mycotoxins exposure, and natural antioxidants can alleviate mycotoxicosis as well as effectively regulate ferroptosis. In recent years, research on the treatment of diseases by Chinese herbal medicine through ferroptosis has attracted more attention. This article reviews the mechanism of ferroptosis, discusses the role of ferroptosis in mycotoxicosis, and summarizes the current status of the regulation of various mycotoxicosis through ferroptosis by Chinese herbal interventions, providing a potential strategy for better involvement of Chinese herbal medicine in the treatment of mycotoxicosis in the future.

## 1. Introduction

Mycotoxins are toxic secondary metabolites produced by a variety of fungi, with characteristics such as enrichment, stability, synergism, and territoriality [1]. Mycotoxins such as aflatoxin (AF), T-2 toxin, zearalenone (ZEN), ochratoxin, and deoxynivalenol (DON, vomitoxin) have drawn much attention due to their serious effects on human and animal health [2]. Aflatoxin mainly affects the liver, kidney, intestine, and immune function, and has the greatest harm to pigs, poultry, and ruminants. Zearalenone is mainly harmful to the reproductive system and can cause abortion in animals, dead fetuses, mummified fetuses, and weak fetuses. The excessive content of zearalenone in feed causes the repeated infertility of sows in many breeding pig farms [2]. The T-2 toxin is mainly produced by *Fusarium graminearum* and can disrupt the lymphatic system, resulting in decreased immunity in animals [3], which also damages the reproductive system, resulting in problems such as reduced egg production with poor eggshell quality and increased egg cleavage rate with thin protein [4]. The gastrointestinal tract is the main target organ for vomitoxin or DON invasion in livestock and poultry which can cause reduced feed intake, refusal to eat, and frequent vomiting, leading to malnutrition and long-term failure. Pocine aflatoxin and fumonisin mainly damage the immune, reproductive, and digestive systems, resulting in decreased immunity, irregular estrus, feed indigestion, digestive tract mucosal damage, and prolonged animal suffering [5]. Moreover, the living environments of fungi are extensive, including grain, feed, and other crops, which are contaminated by fungi mainly in the transportation, storage, processing, and marketing process. Long-term consumption of these feeds by livestock and poultry leads to the accumulation of toxins, resulting in serious damage to some metabolic organs such as the liver and kidneys, and even the risk of meat and dairy contamination increases, causing huge economic losses in the breading industry worldwide [6,7,8]. According to reports, at least 25% of global feed production suffered from mycotoxin contamination, the United Nations Food and Agriculture Organization (US-FAO) estimates that the world’s losses due to mycotoxin contamination, amounting to hundreds of billions of dollars a year [9]. Mycotoxin contamination has become a huge challenge to control in recent years due to global warming, with a higher incidence of mycotoxins in eastern Kenya due to hot and humid weather, whereas cold and dry northern regions such as Canada have the lowest incidence of mycotoxins [10]. If *Aspergillus flavus* (*A. flavus*) continues to grow indefinitely under high temperatures and drought conditions, posing a serious threat to the health of human beings and animals [11].

The common features of fungal toxins in humans and animals mainly include liver, kidney, and intestinal injury, and even immunosuppression, which are partly caused by cellular inflammation, oxidative stress, apoptosis, autophagy, ferroptosis, and other forms of cell death [8,12,13,14,15]. Recent studies showed that mycotoxins can induce liver toxicity, acute kidney injury, or intestinal damage by promoting ferroptosis, manifested by the death of tubule epithelial cells and hepatocytes, and damage to the intestinal barrier function and gut microbial homeostasis [12,15,16]. Interestingly, treatment of cells with the ferroptosis inhibitor ferrostatin-1 significantly restored the toxicity of mycotoxins (such as T-2 toxin) and also found that T-2 toxin triggered ferroptosis by inducing ROS, suggesting that ferroptosis is related to T-2 toxin-related toxicity and could be a potential target for the treatment of mycotoxicosis [17]. In addition, the elevated hepatorenal iron levels and the activation of lipid peroxidation caused by various reasons create pathological conditions for the occurrence of hepatocyte or renal tubular ferroptosis [18,19]. Ferroptosis, a form of cell death typically characterized by iron deposition and lipid peroxidation, was formally proposed in 2012 [20]. It has been demonstrated that ferroptosis is also associated with pathological mechanisms of ischemic and fibrotic damage in multiple organs, including cardiomyocyte injury caused by mycotoxin [21,22,23].

Moreover, with the in-depth study of ferroptosis, more and more evidence presented that ferroptosis is associated with cancer and cardiovascular disease [20,24]. This finding helps to develop a new cell protection strategy to protect cells in cancer and heart diseases by inhibiting ferroptosis. Meanwhile, selective induction of ferroptosis has emerged as a potential therapeutic strategy for some cancers [20]. As cell metabolism determines cell death, researchers have attempted to intervene by interfering with ferroptosis molecules and related signaling cascades to reduce cell damage and delay or reverse cell death based on the adaptability of ferroptosis molecules [25]. Therefore, blocking the ferroptosis process of multiple organs may be an effective way to treat mycotoxicosis.

Considering the universality and severity of mycotoxin contamination, it is vital to seek a safe, green, and efficient method of degradation of mycotoxins. Traditional mycotoxin removal techniques, such as physical adsorption and biological detoxification, can reduce the harm of mycotoxins to some extent [26]. At present, more research has begun to focus on the role of herbal extracts or plant extracts in degrading mycotoxins and reducing their toxicity. This method is environmentally friendly, non-polluting, and effective, and also has the ability to regulate the immune function of animals, promote the repair and regeneration of the liver and other organs, facilitate the bio-neutralization of mycotoxins in the liver, and accelerate the excretion of mycotoxins [12,27]. There is a large number of natural compounds and Chinese herbal medicine resources that are safe and contain few toxins [13]. Chinese herbal medicines contain a variety of effective ingredients including proteins, terpenoids, alkaloids, fats, etc. that possess beneficial effects in multiple channels and targets [28]. Some studies have demonstrated that the active ingredients of Chinese herbal medicine act on fungal cells to kill their activity, resulting in anti-fungal effects, and regulate ferroptosis by exerting antioxidant effect from the perspective of molecular mechanisms, which lay a solid foundation for the intervention of Chinese herbal medicine in mycotoxicosis [28,29]. It is noteworthy, however, that few documents have explored ferroptosis regulation from the perspective of pathogenic mechanisms and targeted regulation in mycotoxin poisoning. This paper briefly introduces the mechanism of ferroptosis and discusses its pathological association with mycotoxin poisoning. In addition, the active ingredients in Chinese herbal medicines/compounds were used as entry points to verify their modulating effects on ferroptosis, and the research status of various Chinese herbal medicines in regulating mycotoxicosis was summarized, which provide an insight to focus on the potential mechanisms of Chinese herbal medicine interventions in mycotoxins such as aflatoxin B1 (AFB1), Zearalenone, T-2 toxin, and DON.

## 2. Mechanisms of Ferroptosis

**Figure 1:**
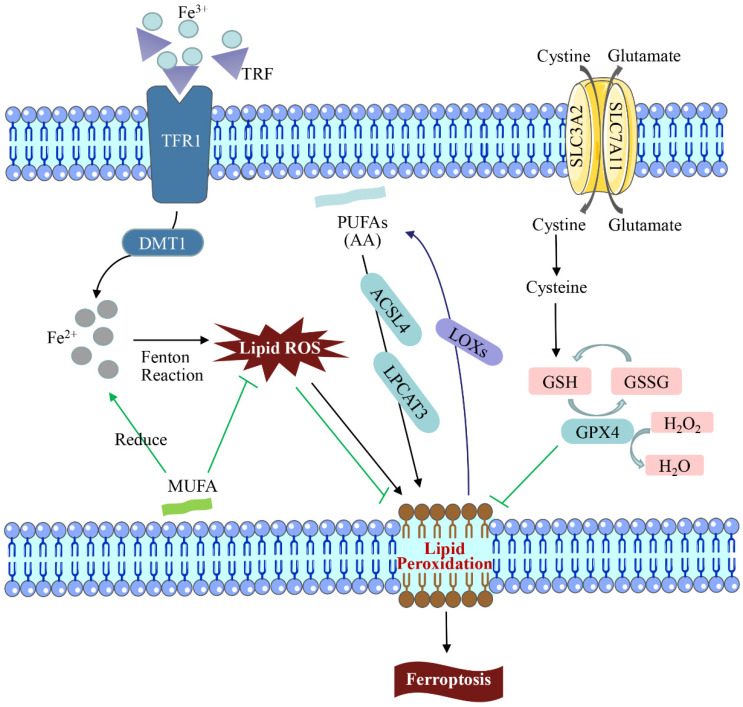
Regulation of the ferroptosis pathway. In the iron cycle, excessive amounts of transferrin (TRF/TF) after binding to trivalent iron (Fe^3+^) may lead to iron overload, catalytic peroxidation, and accumulation of excessive intracellular ROS, resulting in DNA and mitochondrial DNA (mtDNA) damage. Lipid peroxidation (LPO) is the main cause of ferroptosis. LPO attacks PUFAs and expands the oxidation reaction under the action of lipoxygenases (LOXs), causing damage. GPX4 can catalyze the conversion of GSH into GSSG and reduce the intracellular toxic lipid peroxides or free H_2_O_2_ into water. The consumption and activity reduction of GSH and GPX4 in the ferroptosis mechanism led to the decrease in ROS accumulation and LPO scavenging ability, which leads to ferroptosis.

Cell death includes apoptosis, ferroptosis, pyroptosis, lysosome-dependent cell death, autophagy-dependent cell death (autologous death), etc. [30]. Among them, ferroptosis is a non-apoptotic cell death that is different from other death modes in recent years. It is primarily caused by an excessive accumulation of intracellular ROS, impaired iron ion metabolism, and imbalance of cellular lipid peroxidation, which results in decreased glutathione peroxidase 4 (GPX4) activity, elevated intracellular free iron levels, and accumulation of lipid peroxide [31]. As a result of an increase in free iron in a cell, ferroptosis is manifested, which produces more reactive oxygen species and oxidizes polyunsaturated fatty acids on the cell membrane to lipid peroxides via the Fenton reaction. However, it can also catalyze lipid peroxides into toxic lipid radicals, leading to cell death [32]. Ferroptosis has unique cellular morphological, biochemical, and genetic characteristics [33]. Ferroptosis differs from other programmed cell death forms such as apoptosis and pyroptosis in that the morphological changes are driven primarily by changes in mitochondrial structure. Consequently, the volume is reduced, the structural integrity is lost, and the membrane density increases [34]. In 2003, the Stockwell team found that Erastin could induce tumor cell death, and the process was not affected by apoptosis, necrosis, and autophagic cell death inhibitors, showing obvious iron dependence and oxidative dependence [35]. The lipid ROS level in the solute of tumor cells treated with Erastin increased in a time-dependent manner until cell death, which could be effectively blocked by iron chelators and lipophilic antioxidants [36]. It can be seen that excessive accumulation of iron-dependent lipid peroxides becomes the culprit inducing ferroptosis (Figure 1).

### 2.1. Iron Metabolism

Iron is one of the most important metal elements in the animal body and is inextricably linked to the metabolism of the animal body because it is a transition metal that readily gains or loses electrons to participate in redox reactions [37]. The dynamic changes in iron redox enhance the sensitivity of cells to ferroptosis [38]. As a result of inadequate iron in the body, iron deficiency can occur and iron-containing proteins may not be synthesized; too much iron may lead to iron overload, which can cause peroxidation reactions and lead to excessive ROS accumulation within cells, causing DNA and mitochondrial DNA (mtDNA) damage [38,39].

The presence of iron is necessary for the execution of ferroptosis [40]. In the iron cycle, transferrin (TRF/TF) binds to trivalent iron ions (Fe^3+^) and is transported to bone marrow and other tissues in a soluble, non-toxic form [40]. TRF binds to Fe^3+^ with high affinity at physiological pH and is ingested by transferrin receptor 1 (TFR1) on the cell membrane surface. TRF is internalized after binding to TFR1, Fe^3+^ is reduced to Fe^2+^ and released into the cytoplasm via divalent metal ion transporter 1 (DMT1), which can be stored in ferritin or pumped out by a membrane iron transporter [41]. Overloading cells with Fe^2+^ can induce ferroptosis by producing large amounts of ROS via the Fenton reaction [42]. Therefore, regulation of iron import, export, storage, and circular transportation can affect the sensitivity of ferroptosis.

### 2.2. Lipid Metabolism

Lipid metabolism affects the sensitivity of cells to ferroptosis and the degree of ferroptosis [43]. Diallyl hydrogen atoms in polyunsaturated fatty acids (PUFAs) are sensitive to lipid peroxidation and play an important role in ferroptosis [44]. However, monounsaturated fatty acid (MUFA) plays a contrasting role from PUFAs in ferroptosis. PUFAs, such as arachidonic acid (AA), are key substrates for lipid peroxidation (LPO) in ferroptosis, whereas MUFA is structurally resistant to LPO and can inhibit ferroptosis [45]. 

LPO is the primary contributor to ferroptosis. Apart from the Fenton reaction, arachidonyl phospholipid and adrenalacyl phospholipid were catalyzed by acyl-CoA synthetase long-chain family member 4 (ACSL4), lysophosphatidylcholine acyltransferase 3 (LPCAT3) and 15-lipoxygenase to produce LPO [46]. LPO attacks PUFAs and expands oxidative reactions under the action of lipoxygenases (LOXs), causing damage [47]. In addition, ACSL4 is a pivotal enzyme in regulating lipid composition and has been shown to contribute to the execution of ferroptosis [48]. Studies have shown that deficiency of ACSL4 disrupts the homeostasis of lipid metabolism, and knockdown of LPCAT3, a gene involved in phospholipid synthesis, reduces the raw materials for lipid metabolism, both of which increase the resistance of cells to ferroptosis [49].

### 2.3. System Xc-/GPX4 Pathway Regulation

Cell ferroptosis is mainly manifested as decreased glutathione peroxidase 4 (GPX4) activity and glutathione (GSH) depletion at the molecular level [50]. GSH is the most abundant intracellular antioxidant that protects DNA, proteins, lipids, and other biomolecules from oxidative damage [51]. As a selenoprotein that repairs oxidative damage of lipid cells in mammals, GPX4 can catalyze the conversion of GSH to GSSG, and, at the same time, reduce intracellular toxic lipid peroxides to non-toxic hydroxyl compounds or convert free H_2_O_2_ into the water, protecting cell membrane structure and function from interference and damage by peroxides [52]. Therefore, both depletion and reduced activity of GSH and GPX4 can lead to the accumulation of ROS and decreased ability to scavenge LPO, which induces ferroptosis [53].

System Xc- is an important antioxidant system composed of solute carrier family 7 member 11 (SLC7A11) and solute carrier family 3 member 2 (SLC3A2) subunits in the cell membrane, which is essential for the synthesis of glutathione [54]. It can take up extracellular cystine in the cell in a 1:1 ratio and is rapidly reduced to cysteine while pumping out glutamate [55]. Furthermore, a study found that tumor suppressor gene p53 could inhibit the absorption of cystine by System Xc- via down-regulating the expression of SLC7A11, which ultimately triggered ferroptosis. Therefore, GSH, GPX4, and system Xc- are important targets for regulating ferroptosis by medicines [56].

### 2.4. Mitochondrion-Mediated Ferroptosis

Mitochondria are important intracellular organelles that are critical sources of ROS and oxidative stress, and their main function is to provide large amounts of energy through oxidative phosphorylation of the respiratory chain [57]. In addition, mitochondria can also serve as hubs for some metabolic or signaling pathways, especially fatty acid metabolism, which possesses a multifaceted role in mediated ferroptosis [58]. For example, mitochondrial voltage-dependent anion-selective channels (VDAC) are transmembrane channels for transporting ions and metabolites and play an essential role in the regulation of ferroptosis [59]. In mammals, the VDAC family consists of three homologous genes (VDAC1/2/3), and it was found that the ferroptosis inducer Erastin acts on VDAC2 and VDAC3, resulting in mitochondrial dysfunction and consequent release of large amounts of oxides, which ultimately trigger ferroptosis [60]. For another, dihydroorotate dehydrogenase (DHODH) of the mitochondrial inner membrane can resist ferroptosis in mitochondria by regulating CoQH2 production [61]. Furthermore, a study has pointed out that mitochondria may regulate ferroptosis through the tricarboxylic acid (TCA) cycle [58].

## 3. Role of Ferroptosis in Mycotoxicosis Diseases

**Figure 2:**
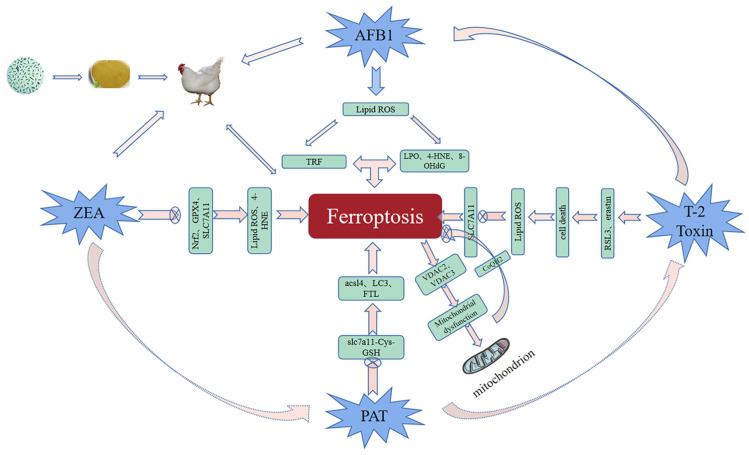
Mycotoxin regulation ferroptosis diagram. The livestock and poultry are fed with feed contaminated by mycotoxins such as aflatoxin, zearalenone, T-2 toxin, and patulin, which promotes the production of ROS, down-regulates the expression levels of ferroptosis-related factors such as SLC7A11, Nrf2, SLC7A11, and GPX4, induces the damage of the biomacromolecular structure and oxidative stress, mitochondrial dysfunction, and leads to ferroptosis.

In recent years, the contamination of mycotoxins has gradually become more serious with global warming, resulting in feed ingredients being contaminated with multiple mycotoxins simultaneously [62]. The common mycotoxins produced are AFB1, ZEN, T-2 toxin, patulin (PAT), etc. According to statistics, more than 25% of the world’s crops are contaminated by mycotoxins every year, increasing the probability and mortality of livestock and poultry infections and causing huge economic losses in agriculture [63]. Mycotoxin contamination has become an urgent problem that threatens human food safety. Due to the cumulative effect of mycotoxins, with the aggregation of the food chain, it has become one of the environmental pollutants that seriously threaten human health [64]. Therefore, it is urgent to explore measures to inhibit mycotoxicosis. The LF, a member of the TRF family, has been reported to have broad-spectrum antibacterial, antioxidant, anticancer, and other biological functions, and it is effective in alleviating fungal toxins as one of the active ingredients of antimicrobial peptides (AMPs) [65]. Ferroptosis is known to be activated by TRF, and in studies of ferroptosis and AFB1, ferroptosis and DON, ferroptosis and ZEN, and ferroptosis and T-2 toxin, it was found that exploring the link between ferroptosis and fungal toxins could contribute to a breakthrough in treating mycotoxicosis (Figure 2).

### 3.1. AFB1 and Ferroptosis

The most toxic and carcinogenic mycotoxin known is AFB1, a secondary metabolite of *A. flavus* and *Aspergillus fungi* (*A. fungi*) displayed that AFB1-induced cardiotoxicity is associated with ferroptosis regulation [22]. AFB1 induced lipid peroxidation and increased the expression of ferroptosis activators (TRF and solute carrier family 11 Member 2) by promoting the production of ROS. At the same time, concurrent exposure to AFB1 promoted the accumulation of LPO and the deposition of 4-hydroxynonenal (4-HNE) and 8-hydroxydeoxyguanosine (8-OHdG), exacerbating hepatocellular carcinoma [66]. Interestingly, exogenous Fe^2+^ can inhibit the growth of *A. flavus* by inducing ferroptosis of *A. flavus* spores [67]. Moreover, MIL-101 (Fe) is a typical iron-containing metal-organic framework that can effectively adsorb AFB1; the iron-containing nanomaterial (PCN-223(Fe)) has good peroxidase-like activity and can sensitively detect AFB1 in milk by constructing an immunosorbent assay [68,69]. Iron therapy has been used to relieve clinical symptoms and improve prognosis in patients with chronic kidney disease [70]. It can be seen that nanomaterials have great potential in the application of AFB1.

### 3.2. Zearalenone and Ferroptosis

Zearalenone (ZEA) is a non-steroidal estrogenic mycotoxin produced by *Fusarium fungi*. Global mycotoxin monitoring reports in the past 10 years have shown that ZEA can be detected in 45% of grains (such as corn, wheat, and soybeans) [1,71]. In 2022, a research team found that ZEN could cause certain damage to mouse sperm, and ferroptosis was involved in this process. This experiment found that the sperm motility and concentration of mice decreased significantly, and the testicular seminiferous tubule structure and antioxidant defense system were also damaged after ZEN exposure, which led to blocked spermatogenesis [72]. Furthermore, it was also noted that ZEN could activate ferroptosis-related signaling pathways and inhibit the expression levels of Nrf2, SLC7A11, and GPX4, resulting in excessive accumulation of lipid peroxides and high expression of 4-HNE protein in mouse testis, while the administration of Ferrostatin-1, an iron shedding inhibitor, at 1.5 mg/kg had the best repairing effect, which was mainly manifested by upregulating the expression of SLC7A11 and GPX4 proteins by upregulating Nrf2 expression, reducing iron accumulation and reversing ZEA-induced ferroptosis [72]. Therefore, paying attention to the mechanism of ferroptosis may provide new insights for alleviating the toxic effects of ZEN on livestock and poultry.

### 3.3. T-2 Toxin and Ferroptosis

The T-2 toxin is the most toxic of type A trichothecenes produced by *Fusarium fungi*. In nature, Fusarium is ubiquitous in barley, wheat, corn, and oats, which can reproduce in large quantities and produce T-2 toxin under suitable conditions [3,4,73]. The physical and chemical properties of T-2 toxin are stable and difficult to remove in the processing of ordinary food and feed, causing great harm to livestock and poultry production by triggering oxidative stress and apoptosis-related pathways [73,74]. A recent study has confirmed that ferroptosis is the result of T-2 toxin-related toxicity and pointed out in detail that T-2 toxin enhanced RAS selective lethal compound 3 (RSL3)-and erastin-induced cell death, but the treatment of ferrostatin-1 significantly restored the sensitization of T-2 toxin, indicating that iron shedding plays an important role in T-2 toxin-induced cytotoxicity [74]. Here, RSL3 and Erastin were used to induce ferroptosis. At the same time, ferrostatin-1, in this experiment, increases lipid ROS levels and down-regulates SLC7A11-induced ferroptosis, proving that ferroptosis is a potential target for treating mycotoxin poisoning [74].

### 3.4. Patulin and Ferroptosis

As a toxic fungal secondary metabolite, patulin is widely found in fruits and vegetables, grains, nuts, and other foods and Chinese herbal medicines. It can enter the body through food intake, skin contact, and other ways, which poses a serious threat to the health of humans and animals [15,17]. Patulin has genotoxicity, immunotoxicity, reproductive toxicity, and other toxicities, which can damage the intestine, kidney, liver, and other organs. The toxic mechanisms of patulin include the induction of biological macromolecular structure damage, induction of oxidative stress damage, induction of autophagy, and destruction of intestinal flora homeostasis [63]. It is widely believed that oxidative stress is a key mechanism underlying ferroptosis in fungal poisoning [71,75]. Some researchers found that patulin promoted rsl3-induced ferroptosis in renal cells by inhibiting the slc7a11-cystine-cysteine-glutathione antioxidant system [76]. Another study confirmed that short-term high-dose intake of PAT resulted in acute kidney injury in mice, and the ferroptosis signaling pathway was also found to be enriched to a higher degree in the assay [15]. Transcriptome sequencing and electron microscopy showed their involvement in iron shedding and autophagy. In addition to inhibiting the antioxidant system, PAT promoted the expression of ACL4, LC3, and ferritin light chain (FTL), leading to an autophagy-dependent iron failure [7]. In particular, co-exposure to fungal toxins exacerbated colonic damage in mice by inducing mitochondrial damage and ferroptosis [16]. In general, these findings will provide a new perspective for finding effective therapeutic approaches under patulin exposure.

## 4. Chinese Herbal Medicine for Ferroptosis-Mediated Mycotoxin Poisoning

Oxidative stress is a state of imbalance between oxidative and antioxidant action, which is a vital factor contributing to aging and disease [77]. When the body cannot resist excessive ROS, a series of immune and metabolic diseases are triggered [78]. In recent years, Chinese herbal medicine has been widely used for the prevention and treatment of various metabolic diseases due to its unique pharmacological characteristics, such as antioxidation, immunomodulatory, anticancer, anti-tumor, and anti-fungal effects. In particular, Fe^2+^ overload causes the accumulation of ROS, which leads to the accumulation of LPO and the induction of ferroptosis [79]. Natural antioxidants in Chinese herbal medicine extracts have the characteristics of safety, green and environmental protection, and lower toxic and side effects than chemically synthesized antioxidants [80]. The active ingredients of common natural antioxidants are mostly flavonoids, polysaccharides, polyphenols, and saponins, which exert antioxidant effects by inhibiting the production of lipid ROS and meanwhile converting H_2_O_2_ into H_2_O [81,82]. In recent years, antioxidant herbs have been widely used to resist mycotoxin exposure and ferroptosis-related diseases, including curcumin, lycopene, fucoidan, and artemisinin [83,84]. 

### 4.1. Regulation of Ferroptosis by Chinese Herbal Medicines

Ferroptosis is caused by lipid peroxidation, and Chinese herbal medicine can be used to treat ferroptosis-related diseases (Table 1). Some researchers showed that fucoidan inhibited iron overload induced by long-term alcohol exposure and protected hepatocytes from ferroptosis [28]. Specifically, fucoidan attenuated alcohol-induced liver oxidative damage in rats by upregulating the p62/Nrf2/SLC7A11 pathway and lowering serum ferritin levels, thereby inhibiting ferroptosis. The environmental pollutant di (2-ethylhexyl) phthalate (DEHP) is a threat to human health. In rats, Dai found that DEHP exposure disrupted iron ion homeostasis, increased lipid peroxidation, and inhibited cysteine/glutamate antiporter, whereas lycopene supplementation dramatically suppressed these ferroptosis characteristics [85].

In addition, Wang et al. [90] indicated that flavonoids could affect iron metabolism and inhibit lipid peroxidation caused by iron overload. Therefore, flavonoids with iron chelation and antioxidant activity may become potential complementary therapies. An interesting study found that mulberry leaf extract morachalcone D exerted a protective effect against Erastin-induced endogenous ferroptosis in HT22 cells, which was associated with the upregulation of glutathione and antioxidant genes [86]. Furthermore, several studies demonstrate that ferroptosis is associated with cancer in multiple organs, which could help develop a new cytoprotective strategy to protect organs by selectively activating ferroptosis of cancer cells [87,91].

### 4.2. Treatment of Mycotoxin Poisoning by Chinese Herbal Medicine

Xu et al. [92] demonstrated that lycopene can attenuate AFB1-induced multi-tissue injury, including liver and spleen. In addition, lycopene was shown to protect against ochratoxin A (OTA)-induced DNA damage and renal cell apoptosis, and markedly reduced renal oxidative stress [93]. Pauletto et al. found that the natural polyphenol curcumin effectively reduced AFB1-induced BFH12 Bovine Fetal Hepatocyte 12 cytotoxicity and decreased cell mortality, mainly due to its powerful antioxidant properties [94].

**Figure 3:**
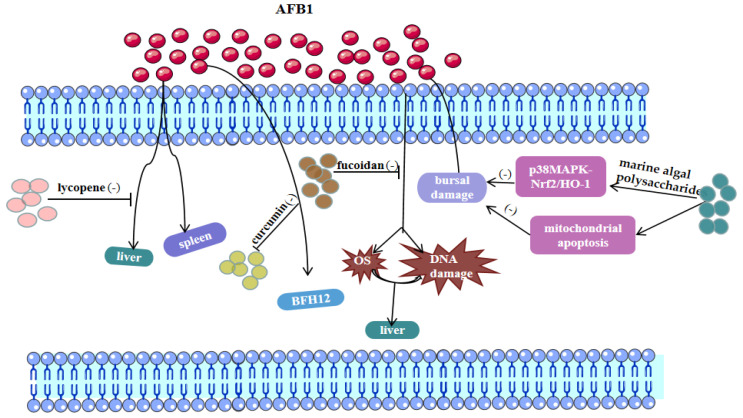
The picture shows the potential mechanisms of lycopene, curcumin, and algal polysaccharides in the treatment of aflatoxin B1. Lycopene mainly reduces aflatoxin damage by affecting the liver and kidneys. Natural polyphenol curcumin can effectively reduce afb1-induced BFH12 toxicity and reduce cell mortality. Algal polysaccharides can mitigate afb1-induced bursal damage in broiler chickens by regulating p38MAPK-Nrf2/HO-1 and mitochondrial apoptosis signaling pathways. AFB1, aflatoxin B1; OS, Oxidative stress; BFH12, Bovine Fetal Hepatocyte 12.

Marine algal polysaccharides and fucoidan are both polysaccharides that exert antioxidant effects [13,95]. Guo et al. [7] showed that marine algal polysaccharides could alleviate AFB1-induced bursal damage in broilers by regulating p38MAPK-Nrf2/HO-1 and mitochondrial apoptosis signaling pathways. Abdel-Daim et al. [96] showed that fucoidan supplementation could inhibit oxidative stress and DNA damage in the liver and kidney of AFB1-exposed rats and increase antioxidant enzyme activity. Milk thistle has the effect of treating liver disease, and its active ingredient ‘silymarin’ from milk thistle belongs to flavonoids. Adding milk thistle to the feed was found to protect the liver from AFB1 damage [97]. Taken together, natural antioxidant active substances inhibiting mycotoxin toxicity have become a research hotspot (Table 2 and Figure 3).

### 4.3. Chinese Herbal Medicine for the Treatment of Mycotoxin Poisoning Mediated by the Ferroptosis Pathway

Lin et al. [16] exposed mice to multiple mycotoxins to induce jejunal mitochondrial dysfunction and oxidative stress-mediated ferroptosis, whereas lycopene ameliorated the impairment of intestinal barrier function from mycotoxins by inhibiting ferroptosis. In rat livers, fucoidan not only exerted a protective effect against AFB1 exposure but also inhibited ferroptosis-induced liver injury, all attributed to the ROS scavenging ability of fucoidan [29,95]. Curcumin, a polyphenolic compound extracted from turmeric, has attracted much attention for its antioxidant and anti-apoptotic activities [48,83,98]. Curcumin attenuated AFB1-induced hepatotoxicity by modulating the LncRNA-mRNA network and Nrf2/HO-1 pathways [21]. In addition, curcumin nanoparticles have been shown to inhibit ferroptosis in hippocampal cells of HT22 mice and inhibit cancer cell development by regulating ferroptosis [88,89]. Overall, natural antioxidants against mycotoxins exposure or acting on ferroptosis may become a trend for future research. However, it remains to be established whether these natural antioxidants can inhibit mycotoxicosis by modulating ferroptosis.

## 5. Conclusions and Perspective Conclusions

Ferroptosis, as programmed cell death, plays an important role in cerebral hemorrhage, ischemic stroke, liver injury, cardiovascular and cerebrovascular diseases, and other multi-system diseases. The expression of iron response element binding protein 2 (IRB2), the main transcription factor that inhibits iron metabolism, can significantly increase the expression of ferritin light chain (FTL) and ferritin heavy chain (FTH1), thus inhibiting ferroptosis induced by erastin. However, mycotoxins such as DON can increase the expression of divalent metal transporter 1 (DMT1) and ferritin FTH1 and FTL, which may be because toxins such as DON mainly promote intestinal iron ion intake and metabolism, and eventually accumulate in tissues, which induces cells to be sensitive to ferroptosis and even triggers ferroptosis. Due to the prevalence of chronic mycotoxin poisoning, the recent research between ferroptosis and mycotoxicosis is mostly biased towards ferroptosis and chronic mycotoxin poisoning, the specific effect of ferroptosis on acute mycotoxin toxicity in the future still needs to be studied and expanded. Therefore, an in-depth study on the mechanism of ferroptosis may be used as a potential target for the treatment of mycotoxicosis.

In the process of production, transportation, and preservation of feed, due to the changes in temperature and humidity in the external environment, it is easy to mildew if it is not handled properly, which not only causes great economic losses to the livestock and poultry industry but also harms the public food safety and human health. Among many hazards of mycotoxins in feed, liver injury is the most common for livestock and poultry. To solve the above issue, Chinese herbal medicine extracts, a potential option, have diversified chemical structures and biological activities, including antioxidant, anti-inflammatory, antibacterial, and antigenic toxicity effects. Therefore, they are widely used in livestock and poultry production and have a good health-care effect on livestock and poultry. In particular, Chinese medicine containing highly volatile oils and high anti-fungal activity has been applied to livestock and poultry feed. The action mechanisms of Chinese herbal medicine in the treatment of mycotoxin poisoning are varied, mainly through oxidative stress, inflammation, liver injury, immunotoxicity, and other paths. Therefore, Chinese herbal medicine can treat mycotoxin poisoning from multiple perspectives and has great drug application value and good application prospects.

In summary, ferroptosis is a key type of oxidative damage in mycotoxicosis and an effective pathway for detoxification. Chinese herbal medicine has accumulated rich experience in inhibiting AFB1 exposure for a long time and therefore is considered a highly promising option in treating mycotoxicosis. At present, the exploration of Chinese herbal medicine for regulating ferroptosis is still in its infancy. However, through the literature review, it is clear that there is still a broad space for the intervention of ferroptosis in liver and kidney cells after mycotoxicosis. In addition, there is a growing consensus that inhibition of ferroptosis and prevention of iron overload may become an effective strategy for treating mycotoxicosis.

## Figures and Tables

**Table 1:** Application of Chinese herbal medicines in ferroptosis.

Chinese Herbal Medicines	Active Ingredient	Organ/Cell	Disease Model/Target	References
Lycopene	Carotenoids	Jejunal tissues	Murine model of multiple-mycotoxin exposure	Lin et al. [16]
Fucoidan	Polysaccharides	Liver tissues	Murine model of alcohol exposure	Xue et al. [29]
Flavonoid 4,4′-dimethoxychalcone	Flavonoids	A549 cells and 786-O cells and 293T cells	Keap1/Nrf2/HMOX1 pathway	Yang et al. [34]
Terpenoid	Artemisinin	Hepatocellular carcinoma cells and glioblastoma cells	GSH or GPX4	Wu et al. [84]
Lycopene	Carotenoids	Spleen tissues	Murine model of DEHP exposure	Dai et al. [85]
morachalcone D	Flavonoids	HT22 cells	Erastin-induced cell death	Wen et al. [86]
Artesunate	Artemisinin	HNC cells	Nrf2–ARE antioxidant signaling pathway	Roh et al. [87]
Curcumin Nanoparticles	Polyphenols	MDCK cells and HT22 cells	Murine model of Intracerebral Hemorrhage	Yang et al. [88]
Curcumin	Polyphenols	Human BC cell lines	Murine model of breast cancer	Cao et al. [89]

**Table 2:** Application of Chinese herbal medicines in mycotoxicosis.

Chinese Herbal Medicines	Active Ingredient	Mycotoxins	Mechanisms	Organ/Cell	References
Marine algal polysaccharides	Polysaccharides	AFB1	p38MAPK-Nrf2/HO-1 and mitochondrial apoptotic signaling pathway	Bursa of Fabricius in broilers	Guo et al. [13]
Curcumin	Polyphenols	AFB1	Upregulation of Nrf2/HO-1 signaling pathway and suppression of ROS and AFB1-adducts production	Liver in chickens	Li et al. [48]
		AFB1	Involve the expression of LncRNAs	Liver in broilers	Li et al. [83]
Lycopene	Carotenoids	AFB1	Enhance antioxidant capacity by activating Nrf2 signaling pathway	Liver in murine	Xu et al. [90]
		AFB1	Enhancement of spleen antioxidant ability and inhibition of lymphocytes apoptosis	Spleen in murine	Xu et al. [91]
Fucoidan	Polysaccharides	AFB1	Restore the hepatorenal markers and enhance the antioxidant enzyme activity	Liver and kidneys in murine	Abdel-Daim et al. [94]
Milk thistle	Flavonoids	AFB1	Restore the hepatic markers	Liver in meat-type chicken	Din et al. [95]
Curcumin	Polyphenols	AFB1	Relate to antioxidant response, defense response, and inflammation	Bovine fetal hepatocytes	Pauletto et al. [96]

## Data Availability

No new data were created or analyzed in this paper. Data sharing is not applicable to this paper.

## Abbreviations

AFB1, aflatoxin B1; ACSL4, acyl-CoA synthetase long-chain family member 4; AA, arachidonic acid; AMPs, antimicrobial peptides; A. flavus, Aspergillus flavus; CAP, composite antimicrobial peptides; DON, deoxynivalenol; DMT1, divalent metal ion transporter 1; DHODH, dihydroorotate dehydrogenase; DEHP, di(2-ethylhexyl) phthalate; FTL, ferritin light chain; GSH, glutathione; GPX4, glutathione peroxidase 4; 4-HNE, 4-hydroxynonenal; 8-OHdG, 8-hydroxydeoxyguanosine; LPO, lipid peroxidation; LPCAT3, lysophosphatidylcholine acyltransferase 3; LOXs, lipoxygenases; LF, Lactoferrin; mtDNA, mitochondrial DNA; MUFA, monounsaturated fatty acid; OTA, ochratoxin; PUFAs, Polyunsaturated fatty acids; PAT, patulin; ROS, reactive oxygen species; SLC7A11, solute carrier family 7 member 11; SLC3A2, solute carrier family 3 member 2; TRF/TF, transferrin; TFR1, transferrin receptor 1; TCA, tricarboxylic acid; VDAC, voltage-dependent anion-se-elective channel; ZEN, zearalenone.
